# Possible Autoimmune Encephalitis with Claustrum Sign in case of Acute SARS-CoV-2 Infection

**DOI:** 10.1017/cjn.2020.209

**Published:** 2020-09-17

**Authors:** Parisa Ayatollahi, Apameh Tarazi, Richard Wennberg

**Affiliations:** Department of Neurology, Shahid Sadoughi Hospital, Yazd University of Medical Sciences, Yazd, Iran; Division of Neurology, Department of Medicine, Toronto Western Hospital, University of Toronto, Toronto, Canada

**Keywords:** COVID-19, Epilepsy, Limbic encephalitis, Magnetic resonance imaging (MRI), Seizure, Steroid immunotherapy

An 18-year-old woman presented to hospital after a 1-week history of fever, fatigue, malaise, and loss of appetite that progressed to include drowsiness and confusion. There was a history of exposure to COVID-19 in a sick family member 6 days prior to symptom onset. The patient had been assessed by an infectious disease specialist and prescribed acetaminophen and levofloxacin. The day before admission she developed urinary retention, and the next day suffered a generalized tonic–clonic seizure.

On admission, temperature was 37.5°, heart rate 92, respiratory rate 15, blood pressure 110/70. A macular rash was evident over the trunk and legs and there was mild suprapubic tenderness. Neurological examination revealed the patient to be drowsy with impaired orientation to time and place. She was able to follow simple verbal commands with normal speech and language. Recent memory was impaired with preservation of remote memory. There was mild diffuse weakness and generalized hyperreflexia, but no lateralizing primary sensorimotor abnormality. Plantar responses were equivocal. She was unable to walk or stand. Within half an hour, she had a second generalized tonic–clonic seizure, lasting 90 s, followed by a postictal state lasting 30 min.

Complete blood count showed thrombocytopenia (89 × 10^9^/L) with normal red and white blood cell counts; normal hemoglobin, mean corpuscular volume, and erythrocyte sedimentation rate. Blood culture, Wright agglutination test, ß-human chorionic gonadotropin, and C-reactive protein negative. Alanine aminotransferase (90 IU), aspartate aminotransferase (55 IU), and creatine phosphokinase (197 U/L) levels were mildly elevated. Electrolytes, glucose, creatinine, blood urea nitrogen, calcium, magnesium, alkaline phosphatase, bilirubin, lactate dehydrogenase (LDH), thyroid-stimulating hormone, complement C3, C4, CH50 levels, and international normalized ratio were normal. Antinuclear, anti-phospholipid, anti-cardiolipin, anti-double-stranded DNA, and antineutrophil cytoplasmic antibodies were negative. Urinalysis was normal. Arterial blood gases were normal (pH 7.39, PaCO_2_ 41.3, PaO_2_ 82.1, HCO_3_^-^ 24.8); oxygen saturation was 95%. Nasopharyngeal reverse transcriptase polymerase chain reaction (PCR) for SARS-CoV-2 was positive.

Brain computed tomography (CT) and magnetic resonance imaging (MRI) scans were normal. Electroencephalography (EEG) showed moderate intermittent nonepileptiform abnormalities (theta/delta slow-wave activity) recorded independently, or occasionally synchronously, over both hemispheres, predominating over the frontocentrotemporal regions bilaterally. Cerebrospinal fluid (CSF) analysis showed normal opening pressure (150 mm H_2_O), increased white blood cells (20 cells/uL, 100% lymphocytes), no red blood cells, normal protein (30 mg/dL), glucose (41 mg/dL), and LDH (28 IU/L) levels. CSF Gram stain and culture negative; CSF PCR for herpes simplex virus negative, CSF PCR for SARS-CoV-2 negative.

Abdominal/pelvic ultrasonography and CT showed mild free fluid in the pelvic cavity and fluid in the endocervical cavity; lung spiral CT revealed no abnormalities.

The patient was empirically treated with intravenous acyclovir on admission and administered intravenous valproate sodium. The next day, she developed myoclonic jerks involving primarily the right side of the face and right leg, occurring in brief runs lasting a few seconds; the jerks decreased and did not involve the leg after increases in valproate sodium dosage. On the third day of admission, new behavioral changes appeared including elated mood, inappropriate laughing, anxiety, and insomnia, leading to treatment with clonazepam, risperidone, and sertraline, in addition to sodium divalproex. She began to complain of chest pain and dyspnea; electrocardiography (ECG) showed inverted T-waves in leads V1–3 with normal echocardiography and negative troponin. By the fourth day of admission, she was experiencing severe generalized pruritis, for which hydroxyzine was started.

She thereafter stabilized, albeit with persistent memory deficits and occasional facial jerks, and was discharged from hospital after 8 days. Six days later, however, she was re-admitted after experiencing two generalized tonic–clonic seizures.

On this second admission, frequent episodes of right-sided or occasionally bilateral lower facial myoclonic or clonic jerks were noted, lasting from a few seconds up to a minute. EEG again showed moderate bilateral nonepileptiform abnormalities. Levetiracetam was prescribed as additional antiseizure treatment. The patient continued to complain of pruritis, anxiety, insomnia, and chest pain. Repeat ECG was unchanged and troponin negative; D-dimer level was elevated (>200 mcg/mL).

**Figure 1:**
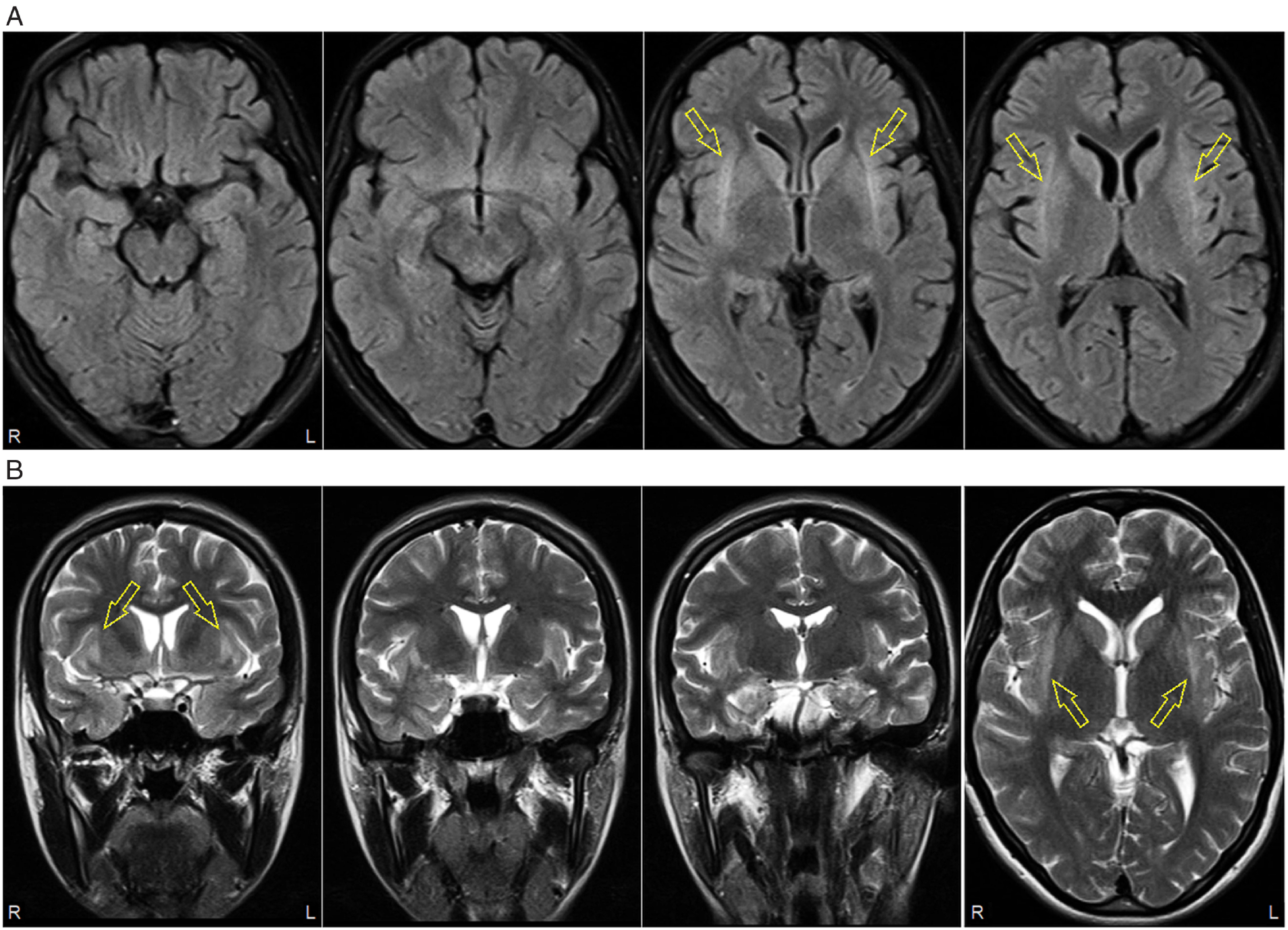
Brain MRI 14 days after initial admission. (A) Axial fluid-attenuated inversion recovery (FLAIR) sequence showing bilateral claustrum hyperintensities (arrows); mesial temporal structures unremarkable. (B) Coronal and axial (far right panel) T2-weighted sequences showing bilateral claustrum hyperintensities extending to external and extreme capsules, with questionable involvement of adjacent insular cortices (arrows).

Repeat brain MRI showed signal hyperintensities on fluid-attenuated inversion recovery (FLAIR) and T2-weighted sequences in the claustrum bilaterally, which were not present on the initial scan 2 weeks earlier. The hyperintensities extended slightly to the external/extreme capsules, with questionable involvement of some adjacent areas of anterior insular cortex; the mesial temporal structures were uninvolved (Figure 1).

Serological testing (tissue indirect immunofluorescence) was negative for the following neural antibodies (N-methyl-D-aspartate receptor, α-amino-3-hydroxy-5-methyl-4-isoxazolepropionic acid receptors, γ-aminobutyric acid B 1/2 receptors, dipeptidyl peptidase-like protein 6, contactin-associated protein-like 2, and leucine-rich glioma inactivated 1). Onconeural antibodies were not screened and CSF was not tested for neural antibodies.

**Figure 2:**
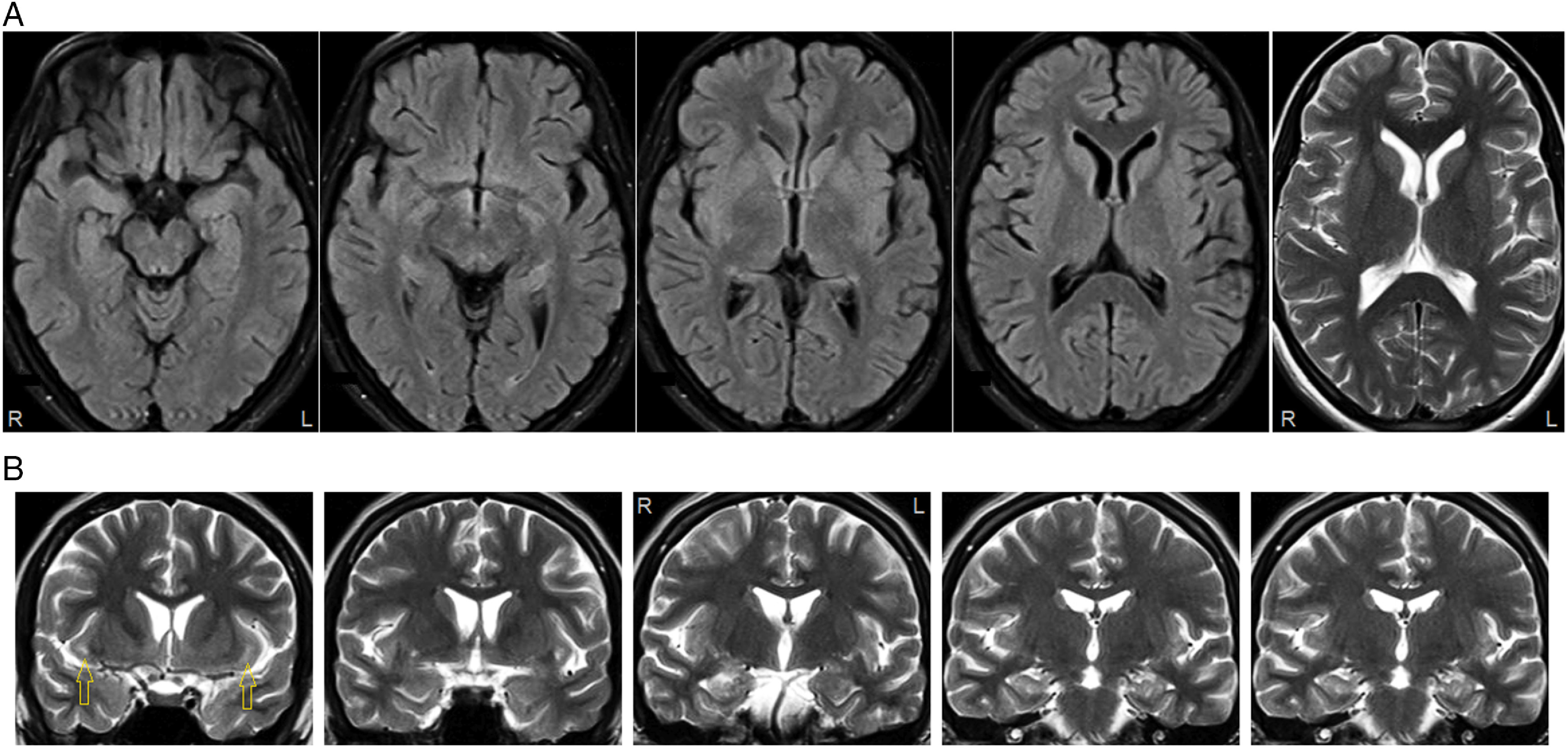
Follow-up brain MRI 1 month after scan shown in Figure 1. (A) Claustrum hyperintensities no longer evident on axial FLAIR or T2-weighted (far right panel) images. (B) Small areas of residual hyperintensity at anterior extent of claustrum and external/extreme capsules (left panel, arrows) on coronal T2-weighted images. Mesial temporal structures unremarkable.

With a diagnostic presumption of “antibody-negative” autoimmune encephalitis,^1,2^ intravenous methylprednisolone pulse therapy was administered (1 g/day for 5 days). On the third day of steroid treatment, the myoclonic jerks stopped, along with improvement of the pruritis and insomnia. She was discharged on clonazepam, sodium divalproex, levetiracetam, and sertraline, with weekly outpatient pulse steroid treatments. Follow-up MRI 1 month after the abnormal scan showed near-complete resolution of the claustrum hyperintensities (Figure 2). Seizures have not recurred (7-week follow-up); recent memory deficits persist.

Claustrum FLAIR/T2 hyperintensities have been proposed as a marker for autoimmune encephalitis/epilepsy,^1^ often appearing after status epilepticus,^1,3^ the encephalitis typically heralded by fever and unassociated with the currently screened autoimmune encephalitis antibodies.^1–3^ The MRI abnormality (“the claustrum sign”) may extend to external/extreme capsules and insular cortices^3^ and typically resolves in weeks or months.^1,3^ Concomitant mesial temporal hyperintensities are present in 33%–50% of patients.^1,3^ The claustrum is one of the most densely connected areas in the brain: the MRI hyperintensities may represent a focused concentration of hyperactivity in epileptic networks connected to the claustrum,^1^ perhaps especially cortical and subcortical areas around the frontal-temporal-opercular region.^3^

Autoimmune encephalitis has rarely been reported as a presentation of COVID-19, and the possibility of a coincidental association in this and other described cases cannot be excluded.^4–7^ The patient described here had only mild non-neurologic symptoms and signs attributable to COVID-19 (fever, malaise, macular rash, urinary retention, and endocervical inflammation); the pruritis was presumed to be of central origin. CSF PCR for SARS-CoV-2 has been nearly invariably negative not only in presumptive COVID-19 encephalitis patients but also in patients with severe systemic COVID-19 illness and neurological/neuroimaging abnormalities, suggesting neurological abnormalities may not be due to glioneuronal infection with SARS-CoV-2.^5,8^ Instead, negative CSF PCR for SARS-CoV-2 may reflect that the underlying neuropathogenesis is autoimmune, which would align with steroid responsiveness. The finding of the claustrum sign on brain MRI, not previously reported in a COVID-19 patient,^4-8^ provides further support for the idea that acute SARS-CoV-2 infection may present as an autoimmune encephalitis.
